# Eculizumab in paroxysmal nocturnal hemoglobinuria with Budd-Chiari syndrome progressing despite anticoagulation

**DOI:** 10.1186/2162-3619-1-26

**Published:** 2012-09-06

**Authors:** Andrés Brodsky, Octavio Mazzocchi, Fabiana Sánchez, Gus Khursigara, Suneil Malhotra, Mariano Volpacchio

**Affiliations:** 1 División Hematología, Hospital de Clínicas “José de San Martin”, Av. Córdoba 2351 (C1120AAR) Ciudad Autónoma de Buenos Aires, 5950-8000 Argentina; 2 Departamento de Medicina, Hospital de Clínicas “José de San Martin”, Av. Córdoba 2351 (C1120AAR) Ciudad Autónoma de Buenos Aires, 5950-8000, Argentina; 3 Diagnóstico por Imágenes, Hospital de Clínicas “José de San Martin”, Av. Córdoba 2351 (C1120AAR) Ciudad Autónoma de Buenos Aires, 5950-8000, Argentina; 4 Alexion Pharmaceuticals, Inc.,, 352 Knotter Drive, Cheshire, CT, 06410, USA

**Keywords:** Budd-Chiari syndrome, Complement inhibition, Eculizumab, Paroxysmal nocturnal hemoglobinuria

## Abstract

Paroxysmal nocturnal hemoglobinuria (PNH) is a progressive, life-threatening disorder characterized by chronic intravascular hemolysis caused by uncontrolled complement activation. Hepatic vein thrombosis (Budd-Chiari syndrome) is common in PNH patients. This case report describes the response to eculizumab (a humanized monoclonal antibody that inhibits terminal complement activation) in a 25-year-old male with progressive liver function deterioration despite standard anticoagulation therapy and transjugular intrahepatic porto-systemic shunt. The patient presented with anemia, severe thrombocytopenia, headache, abdominal pain, and distention. He was diagnosed with PNH, cerebral vein thrombosis, and Budd-Chiari syndrome. Despite adequate anticoagulation, diuretic administration, and placement of a transjugular shunt, additional thrombotic events and progressive liver damage were observed. Eculizumab therapy was initiated, resulting in rapid blockade of intravascular hemolysis, increased platelet counts, ascites resolution, and liver function recovery, all of which are presently sustained. Since starting eculizumab the patient has had no further thrombotic events and his quality of life has dramatically improved. This is the first report to confirm the role of complement-mediated injury in the progression of Budd-Chiari syndrome in a patient with PNH. This case shows that terminal complement blockade with eculizumab can reverse progressive thromboses and hepatic failure that is unresponsive to anticoagulation therapy and suggests that early initiation of eculizumab should be included in the therapeutic regimen of patients with PNH-related Budd-Chiari syndrome.

## Background

Paroxysmal nocturnal hemoglobinuria (PNH) is a life-threatening, progressive, acquired genetic disease characterized by the clonal, nonmalignant expansion of hematopoietic stem cells deficient in glycosylphosphatidylinositol (GPI) synthesis. This deficiency results in fewer GPI-anchored complement inhibitors (CD55 and CD59) on the cell surface, causing increased chronic complement-mediated intravascular hemolysis and platelet hyperactivation and aggregation [1]. Both processes lead to an increased risk of thrombosis, renal dysfunction and damage, pulmonary hypertension, and anemia, which, despite historical treatment regimens, have resulted in up to 35% mortality within 5 years of diagnosis [2]. Thromboembolism is the most common cause of PNH-related death, accounting for two-thirds of all mortalities in patients with this disease [3]. Between 29% and 44% of PNH patients experience a clinically evident thromboembolism, affecting the liver, brain, gut, and kidney [3,4]. Recent registry analyses support an 8.4- to 15.4-fold increased risk of death in patients with PNH with thromboembolism [4,5].

Budd-Chiari syndrome (BCS) is common in PNH patients and anticoagulation therapy is traditionally the first treatment choice for the management of this disorder. However, PNH patients frequently experience new thrombotic episodes despite adequate anticoagulation, which limits the usefulness of subsequent hepatic vein angioplasty and/or stenting and transjugular intrahepatic porto-systemic shunt (TIPS) placement [3,6,7]. Further complicating anticoagulation management, thrombocytopenia occurs in 25% to 52% of PNH patients, creating a high risk of severe bleeding [5,8]. Additional therapeutic options for BCS in patients with PNH are limited to high-risk allogeneic hematopoietic stem-cell transplantation or liver transplantation. In one retrospective study in patients with PNH, there was a 22% reduction in 5-year survival in patients who had received stem-cell transplantation compared with those who had not [9] while liver transplantation in patients with ongoing intravascular hemolysis due to PNH has been associated with high rates of thrombotic and hemorrhagic complications [10].

Eculizumab is a humanized monoclonal antibody that specifically targets the terminal complement protein C5, blocking complement-mediated hemolysis. Two multinational, phase 3 studies and a related extension study demonstrated that eculizumab significantly reduces hemolysis and thrombotic events in patients with PNH [3,11,12]. These studies also showed that eculizumab was effective in reducing renal impairment, pulmonary hypertension, and transfusion requirements, while improving fatigue and quality of life. Furthermore, long-term treatment with eculizumab has been shown to normalize survival of patients with PNH compared with age- and sex-matched controls [6]. Long-term treatment with eculizumab has demonstrated a favorable safety profile [3,11-13].

Here, we describe the effect of eculizumab in a patient with PNH who was being considered for liver transplantation because of multiple thrombotic events, progressive BCS, and declining liver function.

### Case report

**Figure 1:**
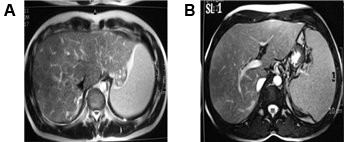
**MRI evidence of BCS.** (**A**) T_2_-weighted MRI. Hepatic veins are not identified. Notice presence of comma-shaped intrahepatic collateral vessels. (**B**) Image obtained caudal to (A) shows a normal appearing portal vein.

**Figure 2:**
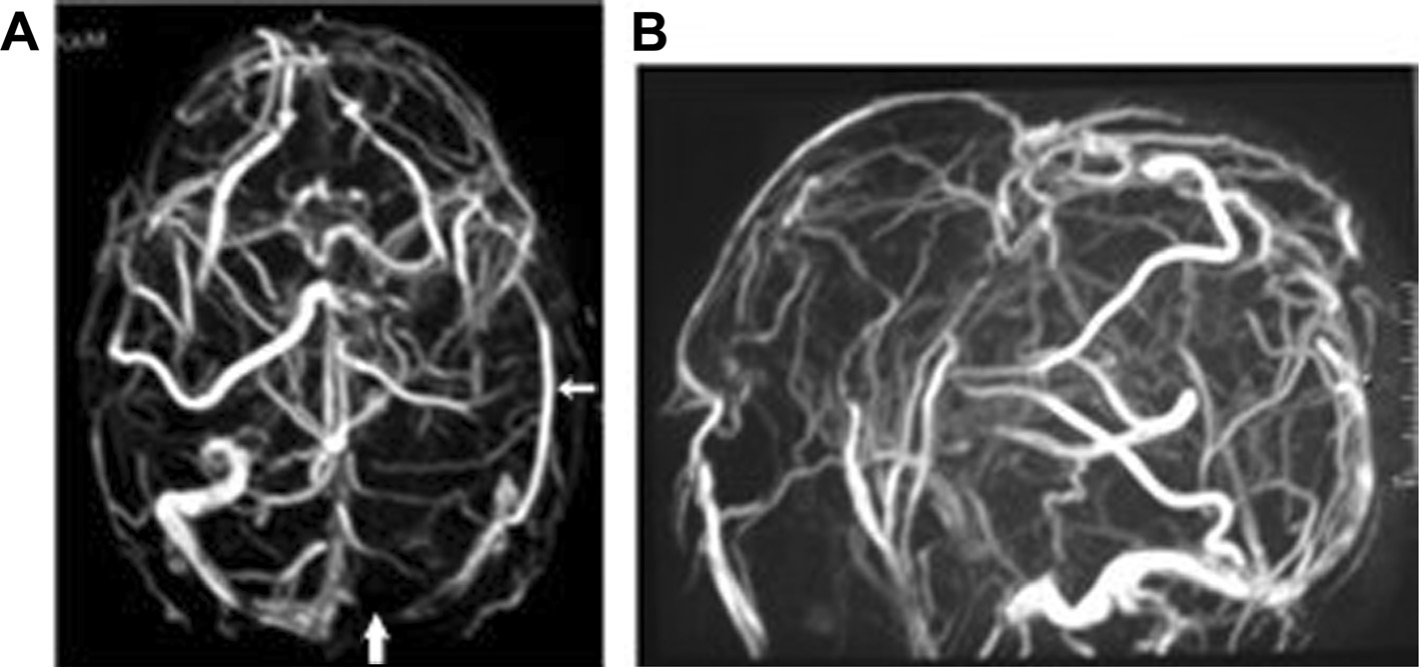
**MRA evidence of cerebral vein thrombosis.** (**A**) A complete thrombotic occlusion of the left transverse sinus vein origin is evident (thick arrow). Collateral circulation (thin arrow) partially fills its proximal portion. (**B**) Partial thrombotic occlusion of the superior sagittal sinus vein.

In June 2005, a 25-year-old white male was hospitalized when he presented with progressive headache, emesis, abdominal pain, and abdominal distension. Ascites was diagnosed and 2500 mL serous liquid was drained via paracentesis. Physical examination revealed hepatomegaly, splenomegaly, and bilateral papilloedema. Blood analysis showed a hemoglobin concentration of 9 g/dL (normal range: 14–18 g/dL), white blood cell count of 3.3 × 10^9^/L (normal range: 4.3–10.8 × 10^9^/L), platelet count of 19 × 10^9^/L (normal range: 150–400 × 10^9^/L), unbound bilirubin concentration of 1.7 mg/dL (normal range: 0.2–0.8 mg/dL), and lactate dehydrogenase (LDH) levels of 1684 U/L (upper limit of normal: 480 U/L). Both Ham and sucrose tests were positive and peripheral blood flow cytometry confirmed the diagnosis of PNH with a granulocyte clone size of 95%. Magnetic resonance imaging (MRI) scans showed BCS (Figure 1) and magnetic resonance angiography (MRA) identified thromboses of the left transverse and superior sagittal cerebral sinus veins (Figure 2).

**Figure 3:**
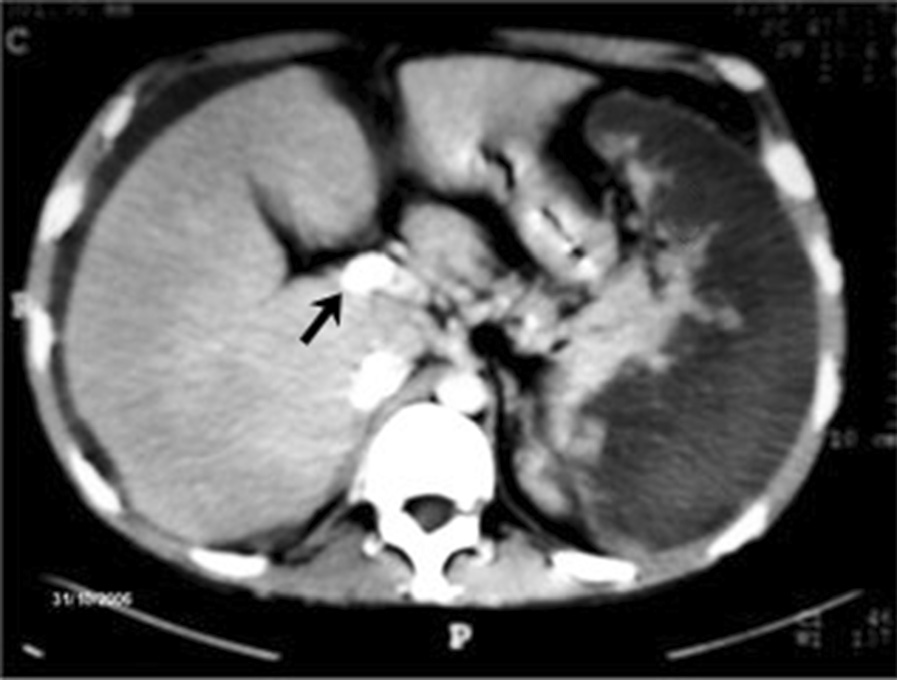
Abdominal computed tomography scan showing a transjugular intrahepatic portosystemic shunt (arrow) and a massive splenic infarct as a result of the splenic embolization.

**Figure 4:**
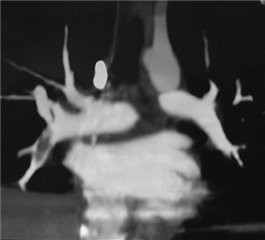
**Coronal reconstruction of a pulmonary computed tomography angiography.** An embolus is seen in the lower right lung artery.

Despite severe thrombocytopenia, the patient received anticoagulation therapy in the form of subcutaneous enoxaparin (1 mg/kg every 12 hours) for 80 months. The patient also received supplemental oral iron and folic acid and was treated with a short course of corticosteroids (methylprednisone, 1 mg/kg/day) to improve thrombocytopenia. In spite of adequate parenteral anticoagulation, BCS progressed and the patient experienced two symptomatic suprahepatic thromboses, increasingly refractory ascites, and painful congestive hepatomegaly, requiring the placement of a TIPS, an immediate splenic embolization (Figure 3), and an additional course of corticosteroids to treat severe refractory thrombocytopenia. A TIPS thrombosis developed and endovascular repermeabilization was required to restore TIPS patency succesfully. A second definitive TIPS thrombosis soon ensued, which was followed by refractory ascites that required paracentesis every 2 to 3 weeks, progressive liver damage (resulting in consideration for liver transplantation), and two episodes of symptomatic pulmonary embolism (Figure 4).

Neither anticoagulation nor TIPS placement prevented further thromboembolisms—the patient experienced two hepatic thromboses and two pulmonary embolisms over more than 40 months of therapy—that increased the extent of liver damage. Therefore, alternative treatment to prevent complement-mediated thrombosis was explored.

In January 2009, eculizumab (Alexion Pharmaceuticals, Cheshire, CT) treatment was initiated at the approved dosing regimen: 600 mg weekly for the first 4 weeks, followed by 900 mg for the fifth dose 1 week later, then 900 mg every 2 weeks thereafter. Within 2 weeks of starting treatment with eculizumab, LDH levels had decreased by 73% from an average of 2.9-fold above normal 1 to 2 months before treatment to within normal levels (≤480 U/L). This reduction was maintained for more than 2.5 years (921 days; Table 1), the latest data collected for this patient. Platelet count increased from 23 × 10^9^/L on day 1 of eculizumab treatment to 53 × 10^9^/L, a 130% increase, after 1 week and was sustained at a level of between 53 and 94 × 10^9^/L (average 67 × 10^9^/L) for 921 days. Rapid improvements in hepatic function were also observed, with alanine aminotransferase and aspartate aminotransferase levels reduced by 68.3% and 76.9%, respectively, from assessments made 68 days prior to treatment initiation to after 1 week of eculizumab therapy (Table 1). The significant reductions in both of these enzymes were sustained over the 921-day treatment time frame reported in this case study. Over the same period, total bilirubin level declined by between 36.1% and 61.1%. The concentration of cholinesterase, a marker of liver function, improved to within the normal level within 56 days of treatment initiation, the earliest time at which this parameter was measured, with a progressive increase thereafter, which is a clear sign of ongoing hepatic functional recovery. Changes in albumin levels paralleled those for cholinesterase and are representative of improvements in the synthetic capacity of the liver.

**Table 1:** Effect of eculizumab on liver function

**Days post-eculizumab**	**Laboratory value (normal range)**								
	**LDH**	**Platelets**	**Hemoglobin**	**AST**	**ALT**	**Bilirubin total**	**Bilirubin D**	**Cholinesterase**	**Albumin**
	**U/L**	**× 10**^**9**^**/L**	**g/dL**	**U/L**	**U/L**	**mg/dL**	**mg/dL**	**U/L**	**g/dL**
	**(≤480)**	**(150–400)**	**(14–18)**	**(≤40)**	**(≤37)**	**(0.2–1.3)**	**(<0.3)**	**(>4400)**	**(3.5–5.0)**
-68	1836	34	9.2	63	78	3.6	1.7	3572	─
-33	921	27	9.8	49	70	2.4	0.9	2889	3.47
0 (1st dose)	─	23	10.7	36	24	3.3	1.4	─	─
7	519	53	9.7	20	18	2.3	1	─	3.54
14	368	57	10.3	15	14	1.9	0.7	─	─
56	565	80	9.6	28	9	2.2	1	5135	3.6
119	248	53	9.9	16	8	1.7	0.8	5975	─
202	295	94	11.3	25	20	2.1	0.9	6968	4.52
300	344	62	10.3	28	25	1.6	0.6	7603	4.68
365	298	58	10.2	22	19	1.6	0.7	─	4.27
449	354	69	11.3	26	26	1.9	0.8	7704	5.1
547	340	63	12	25	24	1.6	0.6	7852	4.73
631	285	73	11.6	26	27	1.8	0.7	8360	─
743	351	65	11.5	26	23	1.7	0.7	8294	4.46
827	388	64	11.2	26	22	1.4	0.6	─	─
921	423	77	12.6	28	25	1.9	0.6	─	4.49

*ALT*, alanine aminotransferase; *AST*, aspartate aminotransferase.

**Figure 5:**
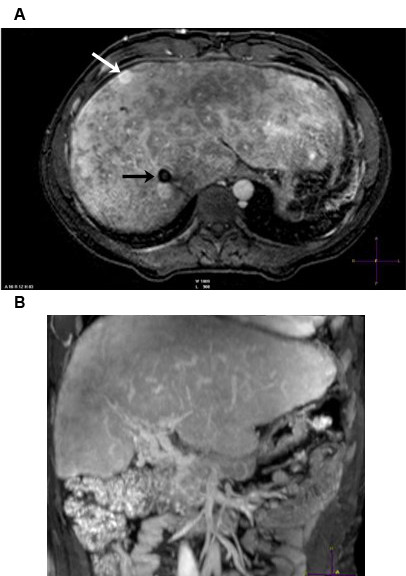
**Contrast-enhanced T**_**1**_**-weighted MRI.** (**A**) The axial view shows morphological changes and heterogeneous enhancement typical of BCS. A hypervascular nodule is seen consistent with a regenerative nodule (white arrow). A TIPS also is seen (black arrow). Notice collateral circulation in the abdominal wall. (**B**) The coronal view shows prominent tortuous vascular structures compatible with cavernomatous transformation of the portal vein.

**Figure 6:**
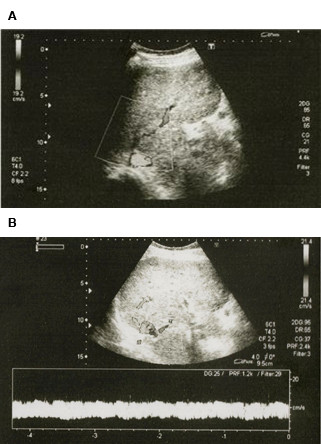
**Sonographic images showing improved hepatic venous flow.** (**A**) Ultrasonographic detection of partial recanalization of the right suprahepatic vein (“narrow pass image”). (**B**) Ultrasound Doppler evaluation of the right suprahepatic vein showing a continuous flow instead of a pulsatile signal. This is due to a proximal partial obstruction of the hepatic venous outflow.

**Figure 7:**
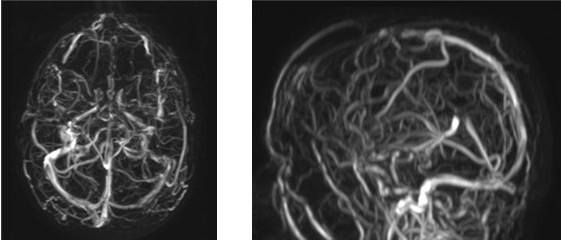
Axial and sagittal venous MRA show resolution of left transverse sinus occlusion and partial resolution of sagittal venous sinus thrombosis.

The interval between paracentesis increased progressively, giving a clear indication of a decrease in portal hypertension. After 7.5 months of eculizumab treatment, no further ascitic fluid evacuation was required. Diuretics, beta blockers, and hydrosaline restriction were also stopped after 2 years of eculizumab treatment. Serial liver MRA studies showed abundant intrahepatic venous collateral circulation without recanalization of the hepatic veins (Figure 5), and development of portal cavernomatosis (evidence of portal vein thrombosis, which appeared after TIPS thrombosis; Figure 5), paralleled the progressive improvement in liver function and hemodynamics. An ultrasound study showed a partial recanalization of the suprahepatic veins, which was unnoticed in MRA studies due to the low blood flow in the recovered blood vessels (Figure 6). In parallel with the improvement in liver vein thrombosis, a near-complete recanalization of the cerebral left transverse venous sinus, as well as partial recanalization of the sagittal vein sinus, also took place (Figure 7). As a consequence of these improvements, plans for liver transplantation were rescinded.

Following initiation of eculizumab, the patient experienced no new symptomatic thrombotic episodes, had a dramatic improvement in fatigue, and did not experience any clinically significant treatment-related adverse events. Consequently, the patient was able to resume a near-normal work and social life.

## Discussion

This is the first report to confirm the role of complement-mediated injury in the progression of BCS in a patient with PNH and its improvement with eculizumab treatment.

Thromboembolism is the most common cause of PNH-related death, accounting for two-thirds of all mortalities in patients with this disease [3]. Between 29% and 44% of PNH patients experience a clinically evident thromboembolism, which affects the liver, brain, gut and kidney [3,4]. This patient was no exception as he developed 2 hepatic and 2 pulmonary thromboembolisms, despite 40 months of anticoagulation and TIPS therapy. Recent registry analyses support an 8.4- to 15.4-fold increased risk of death in patients with PNH with thromboembolism [4,5]. Historically, this increased risk of thrombosis, along with other life-threatening complications such as chronic kidney disease, pulmonary hypertension and anemia, have resulted in up to 35% of patients with PNH dying within 5 years of diagnosis [2].

The patient is currently receiving eculizumab and since starting this therapy has not experienced any new thromboses. This is consistent with recent large cohort analyses in the United Kingdom that show that anticoagulation for primary antithrombotic prophylaxis can be safely discontinued in some patients with PNH who are concurrently receiving eculizumab [6].

Hepatic dysfunction and deterioration in patients with PNH may also result, in part, from increased vascular resistance and/or inflammation, as reported in renal and pulmonary systems [14,15]. For example, chronic complement activation in PNH can lead to local damage of hepatic and/or Kupffer cells, subsequent upregulation of adhesion molecules, and release of proinflammatory cytokines, such as interleukin-1 and -6 [16,17]. In addition, complement-mediated depletion of the smooth muscle relaxant nitric oxide has been linked to pulmonary and systemic hypertension [15,18], as well as reduction of renal blood flow and function [14]. These effects are ameliorated by the inhibition of terminal complement activation by eculizumab, which improves chronic kidney disease in patients with PNH as early as 12 weeks post-treatment [14] and reduces the risk of systemic and pulmonary hypertension [15]. Thus, it is reasonable to suggest that terminal complement inhibition, besides blocking thrombosis in PNH, may prevent vasoconstriction and inflammation that affects both hepatic flow and liver function in PNH patients with BCS. In addition, patients with PNH who are thrombocytopenic, including the patient in this case, are at higher risk of thromboembolism than PNH patients with normal platelet levels [19]. Eculizumab-mediated improvement of platelet count reduces the risk of thromboembolism, and may increase the safety of concomitant anticoagulation and/or subsequent treatment modalities, including allogeneic hematopoietic stem-cell transplantation or liver transplantation.

In this case, the patient responded to eculizumab with a dramatic restoration of hepatic function. The retraction of a planned liver transplant highlights the remarkable improvements that this patient experienced, and bodes well for future PNH cases with similar symptom presentations.

**Figure 8:**
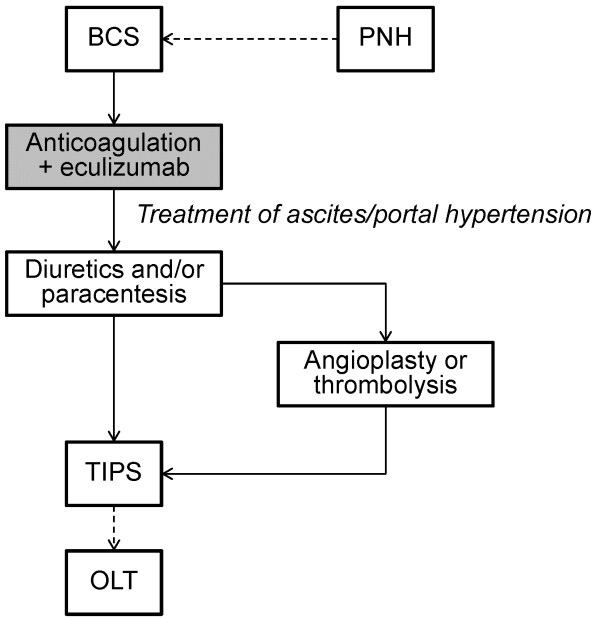
**Revised treatment recommendations for patients with BCS and/or PNH.** As soon as it is established that BCS is secondary to PNH, eculizumab should be considered in the treatment paradigm for these at-risk patients (shaded box indicates proposed revision to the original Hoekstra algorithm). Adapted from Hoekstra J, et al. *J Hepatol*, 2009, with permission from Elsevier [20]. OLT, orthotropic liver transplant.

Eculizumab represents the first treatment for PNH that effectively inhibits complement-mediated intravascular hemolysis and prevents subsequent morbidities, including thromboembolism, renal dysfunction, hypertension, and poor quality of life. Based on the successful treatment of this patient with PNH and BCS, together with the antithrombotic and also the antihypertensive and anti-inflammatory benefits of inhibiting terminal complement activation, we suggest that the treatment algorithm proposed by Hoekstra et al. [20] should be modified to include terminal complement inhibition with eculizumab as a primary therapeutic intervention in PNH patients who present with BCS (Figure 8).

## Conclusions

Eculizumab, a humanized monoclonal antibody that targets the C5 complement protein, is effective at blocking complement-mediated hemolysis in PNH patients. The ability of eculizumab to reverse the damaging effects of BCS confirms the role of complement in the progression of this disease in PNH patients. The absence of new thromboses in this patient after receiving eculizumab supports the application of eculizumab in the treatment of PNH patients with BCS.

### Consent

Written informed consent was obtained from the patient for publication of this case report and any accompanying images. 

## Abbreviations

PNH: Paroxysmal nocturnal hemoglobinuria; GPI: Glycosylphosphatidylinositol; BCS: Budd-Chiari syndrome; TIPS: Transjugular intrahepatic porto-systemic shunt; LDH: Lactate dehydrogenase; MRI: Magnetic resonance imaging; MRA: Magnetic resonance angiography.

## Competing interests

Andrés L. Brodsky: consultancy and speakers bureau for Alexion Pharmaceuticals, Inc. Octavio Mazzocchi: none to declare. Fabiana Sanchez: none to declare. Gus Khursigara: employed by Alexion Pharmaceuticals, Inc., and owns equity in the company. Suneil Malhotra: employed by Alexion Pharmaceuticals, Inc., and owns equity in the company. Mariano Volpacchio: none to declare. Funding assistance for the preparation of this manuscript was provided by Alexion Pharmaceuticals, Inc.
